# Imagining and constraining ferrovolcanic eruptions and landscapes through large-scale experiments

**DOI:** 10.1038/s41467-021-21582-w

**Published:** 2021-03-17

**Authors:** A. Soldati, J. A. Farrell, R. Wysocki, J. A. Karson

**Affiliations:** 1 Department of Marine, Earth, and Atmospheric Sciences, North Carolina State University, Raleigh, NC USA; 2 Department of Earth Sciences, Syracuse University, Syracuse, NY USA; 3 School of Art, Syracuse University, Syracuse, NY USA

**Keywords:** Volcanology, Volcanology

## Abstract

Ferrovolcanism, yet to be directly observed, is the most exotic and poorly understood predicted manifestation of planetary volcanism. Large-scale experiments carried out at the Syracuse Lava Project offer insight into the emplacement dynamics of metallic flows as well as coeval metallic and silicate flows. Here, we find that, under the same environmental conditions, higher-density/lower-viscosity metallic lava moves ten times faster than lower-density/higher-viscosity silicate lava. The overall morphology of the silicate flow is not significantly affected by the co-emplacement of a metallic flow. Rather, the metallic flow is largely decoupled from the silicate flow, occurring mainly in braided channels underneath the silicate flow and as low-relief breakouts from the silicate flow front. Turbulent interactions at the metallic-silicate flow interface result in mingling of the two liquids, preserved as erosional surfaces and sharp contacts. The results have important implications for the interpretation of possible ferrovolcanic landscapes across our solar system.

## Abstract (web summary)

Ferrovolcanism is a hypothetical form of planetary volcanism in which the erupted lava is metallic in composition. Here we show that ferrovolcanic lava is denser and less viscous than silicate lava, resulting in fast-moving, thin, braided flows.

## Introduction

Planetary volcanism can be described as the process whereby a magma, defined specifically for each body based on meltable constituents present, is erupted onto the surface^1^. Solid bodies in the solar system can have one or a combination of three main compositions: rocky (silicate, carbonate), icy (e.g., brines, hydrocarbons), or metallic (alloys). The variety of crustal and mantle materials existing across the solar system should be reflected in the variety of volcanism observed on planetary bodies.

Beyond Earth, silicate volcanism occurred at some point on all terrestrial planets (e.g., Venus, Mars) and some satellites (e.g., the Moon, Io)^2^. Cryovolcanism has been observed (e.g., Enceladus) or inferred (e.g., Europa, Titan, Charon) on many icy worlds^3^. At present, no conclusive evidence has been found for ferrovolcanism on metallic asteroids or planets with a metallic core; however, this type of volcanic activity has been predicted for some planetary bodies^4,5^, raising questions concerning its emplacement processes and the resulting landscapes. The substantiation of ferrovolcanism would complete the range of anticipated volcanism across the solar system.

Volcanic materials across the solar system display a wide range of physical properties and flow dynamics in their respective planetary contexts^3,6,7^. Resulting volcanic landscapes reflect these properties and can provide important clues about the nature of volcanism and the structure and evolution of the planetary bodies upon which they are found.

Ferrovolcanism is a novel concept, and many of its driving parameters are completely unconstrained. Two of the key parameters controlling both silicate volcanism and cryovolcanism are density and viscosity, which play fundamental roles in determining eruptive style and resulting landforms. By analogy, we anticipate similar controls for ferrovolcanism. Therefore, in this study, we comparatively analyze the emplacement dynamics and surface morphologies of coeval silicate and metallic flows through the lens of their contrasting densities and viscosities.

There are at least two conceivable forms of ferrovolcanism: (1) type I: pure ferrovolcanism, taking place on entirely metallic bodies where the conditions for melting and melt emplacement at the surface exist, and (2) type II: spurious ferrovolcanism, occurring on silicate-metallic bodies as the consequence of either (a) melting of the metallic portion or (b) unmixing of an iron-rich silicate melt.

16 Psyche, situated in the asteroid belt between Mars and Jupiter, is the largest (250 km in diameter) M-type asteroid^8^. A primarily iron-nickel surface composition is compatible both with infrared interferometric observations^9^ and radar albedo observations^10^. It is likely that as 16 Psyche cooled, relatively low-density molten metallic material in the interior would have erupted onto its surface in type I ferrovolcanism^4,5^. Interest in 16 Psyche has resulted in a NASA space mission concept^11^ which has been selected for development and is currently scheduled to launch in 2022. Instrumentation on this space orbiter will include a multispectral imager that will provide detailed views of its surface. Images from this mission will shed the first light on this kind of possible metal worlds. However, interpreting those data will be challenging, as there are no known terrestrial analogs to provide guidance.

Active ferrovolcanism has never been observed, but observations have been made of the geomorphological features potentially associated with type IIb ferrovolcanism on Earth. A prime example is the magnetite flows of El Laco, Chile, described and studied by Keller et al. (2019)^12^. Several hypotheses have been made to explain these deposits: one maintains that they formed through metasomatism^13^, and yet another suggests that they formed through magmatic flotation of bubble-oxide aggregates^14^. Keller et al. (2019)^12^ suggested that the El Laco magnetite deposits formed due to the unmixing of a parent silicate liquid into an iron-rich and an iron-poor melt. The higher-density iron-rich melt would have segregated and pooled underneath the iron-poor melt^15–17^, until its tectonic stress-enabled extrusion onto the surface^12^. This genetic mechanism is effectively ferrovolcanism (type IIb).

In this paper, we describe large-scale experiments emplacing coeval silicate and metallic flows. We apply the insight gained from our large-scale experiments to understanding the contrasting emplacement dynamics and final morphologies of mixed silicate and metallic lava flows (ferrovolcanism type IIb), and to get preliminary insight on the flow behavior and morphology of metallic lava flows (ferrovolcanism type I). We show that the density and viscosity contrast between silicate and metallic lava results in largely decoupled emplacement dynamics. Furthermore, metallic lava flows ten times faster than silicate lava, and is characterized by a low-relief, braided morphology.

## Results

### Flow emplacement

Mixed silicate-metallic flows are routinely produced at the Syracuse Lava Project. Typically, the furnace remains active for at least one week, during which time multiple silicate experimental flows are emplaced daily by tilting and partially emptying the crucible. On the last pour before turning off the furnace, the crucible is emptied completely. On these final pours, metallic flows are commonly produced. Here we describe in detail a well-documented experiment (flow 170410; Supplementary Movie 1) in which silicate and metallic flows were co-emplaced, analogous to what might be expected in type IIb ferrovolcanism.

**Fig. 1:**
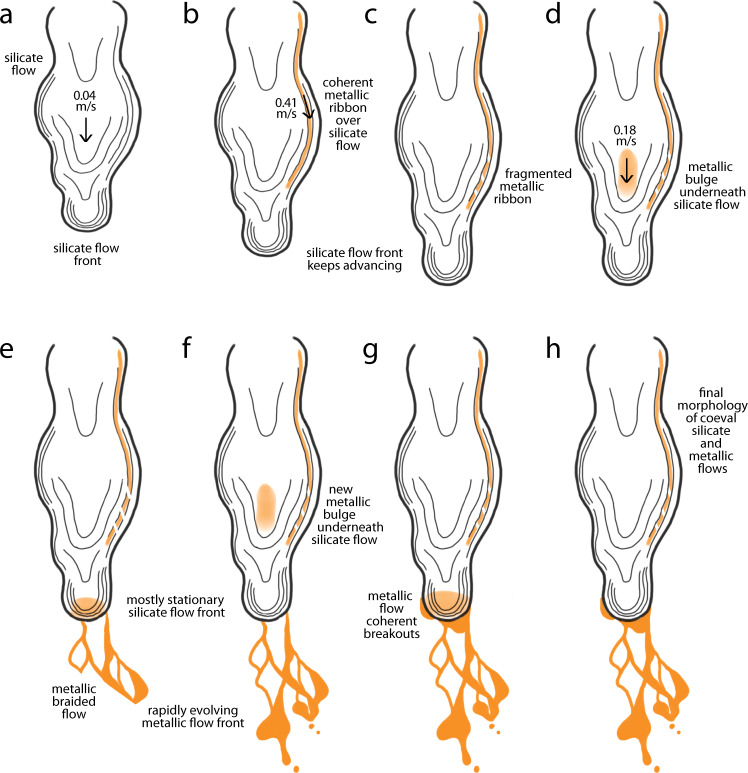
Flow emplacement. **a** Silicate sheet, ropey flow advancing at 0.04 m/s. **b** Metallic ribbon (yellow) emplaced at 0.41 m/s over the silicate flow, following its ropey surface texture; **c** metallic ribbon being dismembered as the surface of advancing silicate flow deforms. **d** Metallic bulge (yellow patch) traveling underneath the silicate flow, with mixed-flow velocity of 0.18 m/s. **e** Metallic bulge reaching the silicate flow front and violently breaking out from it, emplacing a braided metallic flow; **d**, **e** are repeated. **f** Third metallic pulse traveling underneath the silicate flow. **g** Last metallic bulge gently breaking out from the silicate flow front emplacing a few coherent metallic breakouts. **h** Final morphology of the co-emplaced silicate and metallic flows: the silicate flow is a coherent, ropey sheet, whereas the metallic flow body is partly lumpy and cohesive and partly braided and not fully coherent; the metallic flow front extends past the silicate flow front.

Initially, a typical experimental silicate flow was established (Fig. 1a). The flow velocity of the silicate lava was around 0.04 m/s. When the crucible was tilted further and emptied completely, we observed molten metal issuing from the spout, accompanied by sparks. The metallic lava advanced rapidly at a speed of 0.41 m/s, traveling in narrow streams over and under the silicate lava.

At this point, the silicate flow had already come to an almost complete halt. Some metal traveled over the silicate flow in narrow (cm-wide), thin (mm-thick) streams that followed the fine-scale topography of the ropey surface of the underlying silicate flow (Fig. 1b). These silvery metallic ribbons cooled rapidly, despite the heat of the underlying lava, and although they were coherent during flow (Fig. 1b), underlying silicate flow deformation resulted in fragmentation and local dismemberment of the frozen metallic mini-channels (Fig. 1c).

Most of the denser molten metal sank through the still partially molten silicate flow and traveled underneath it. Although we could not directly observe this process in real time, three successive inflation bulges, which we infer to be caused by the metallic melt, deformed the surface of the silicate flow and quickly progressed downslope with a velocity of 0.18 m/s (Fig. 1d). The metal became visible again after a few seconds as it quickly overtook the basaltic flow, it accumulated near the flow front, and eventually broke out from the front of the silicate flow at high speed (Fig. 1e). We observed three metal breakouts from the silicate flow front, spaced about 10 s apart. The first two lasted 3 s each and were violent (Fig. 1d, e), whereas the last was a gentle, more prolonged (25 s) extrusion (Fig. 1f, g). During this final part of the emplacement process, the metallic flow proceeded independently from the silicate flow, thus providing insight on the possible flow dynamics of type I ferrovolcanism.

### Flow morphology

**Fig. 2:**
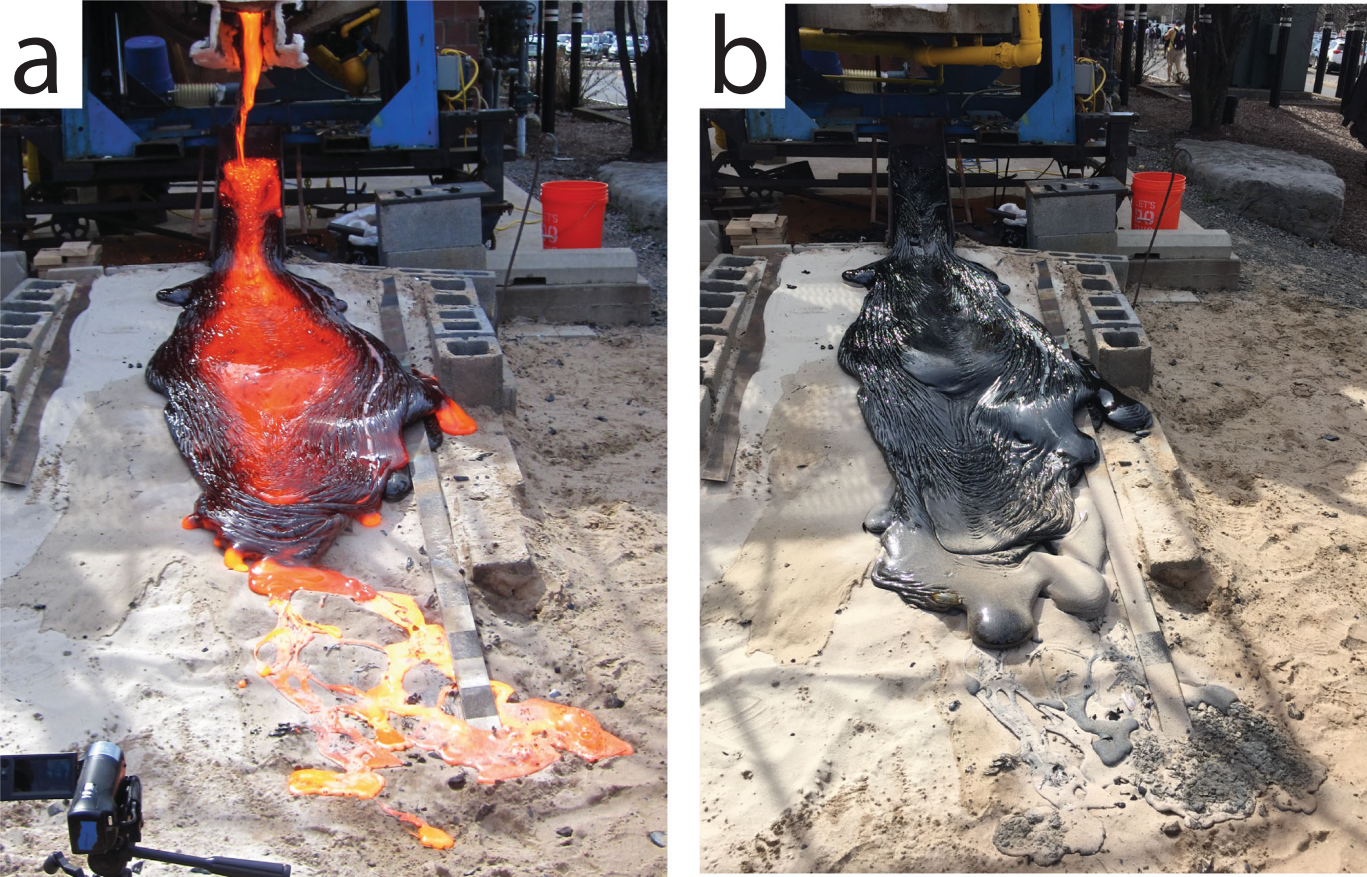
Flow morphologies. **a** Syn-emplacement we observe the metallic flow (yellow) emerging from underneath the silicate flow (orange-black). **b** Post-emplacement the metallic flow appears gray/silver, whereas the silicate flow is black.

**Fig. 3:**
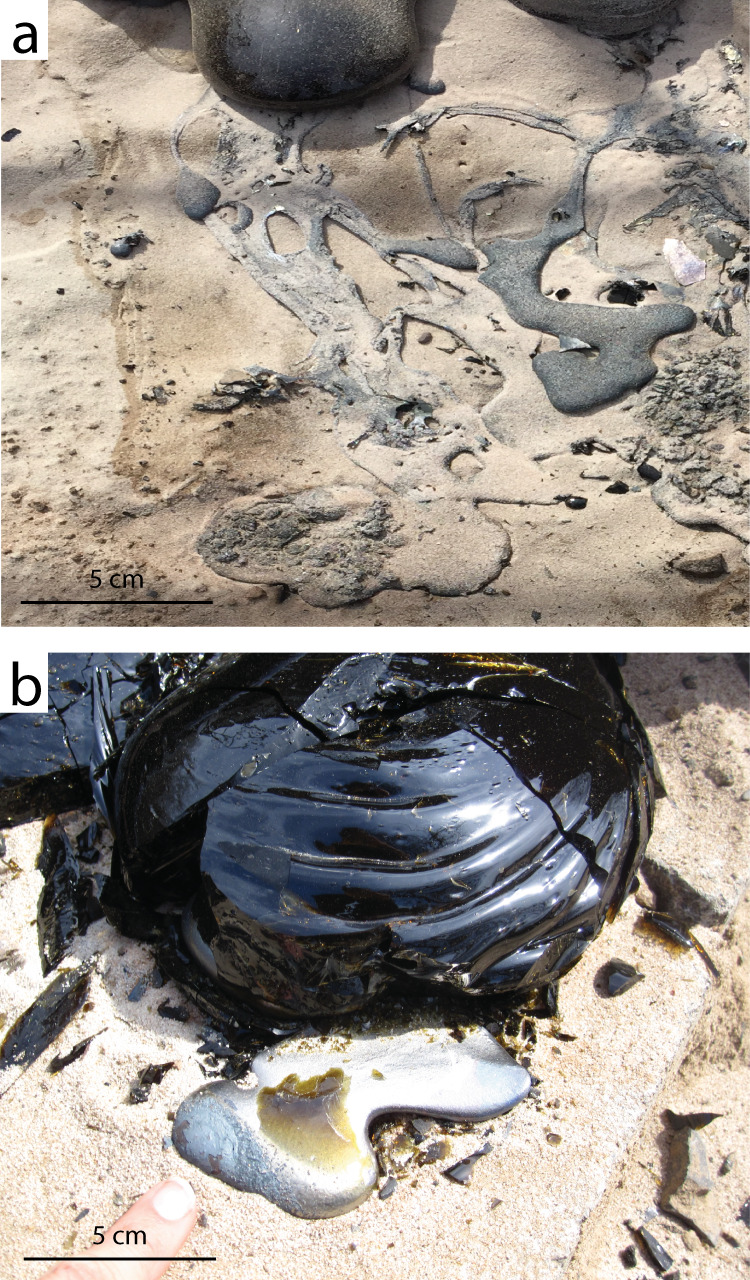
Metallic flow breakout morphologies. **a** Early metallic flow braided breakout (silver) from underneath the silicate flow front (black). **b** Late metallic flow cohesive breakout (silver) from the silicate flow (black) front.

**Fig. 4:**
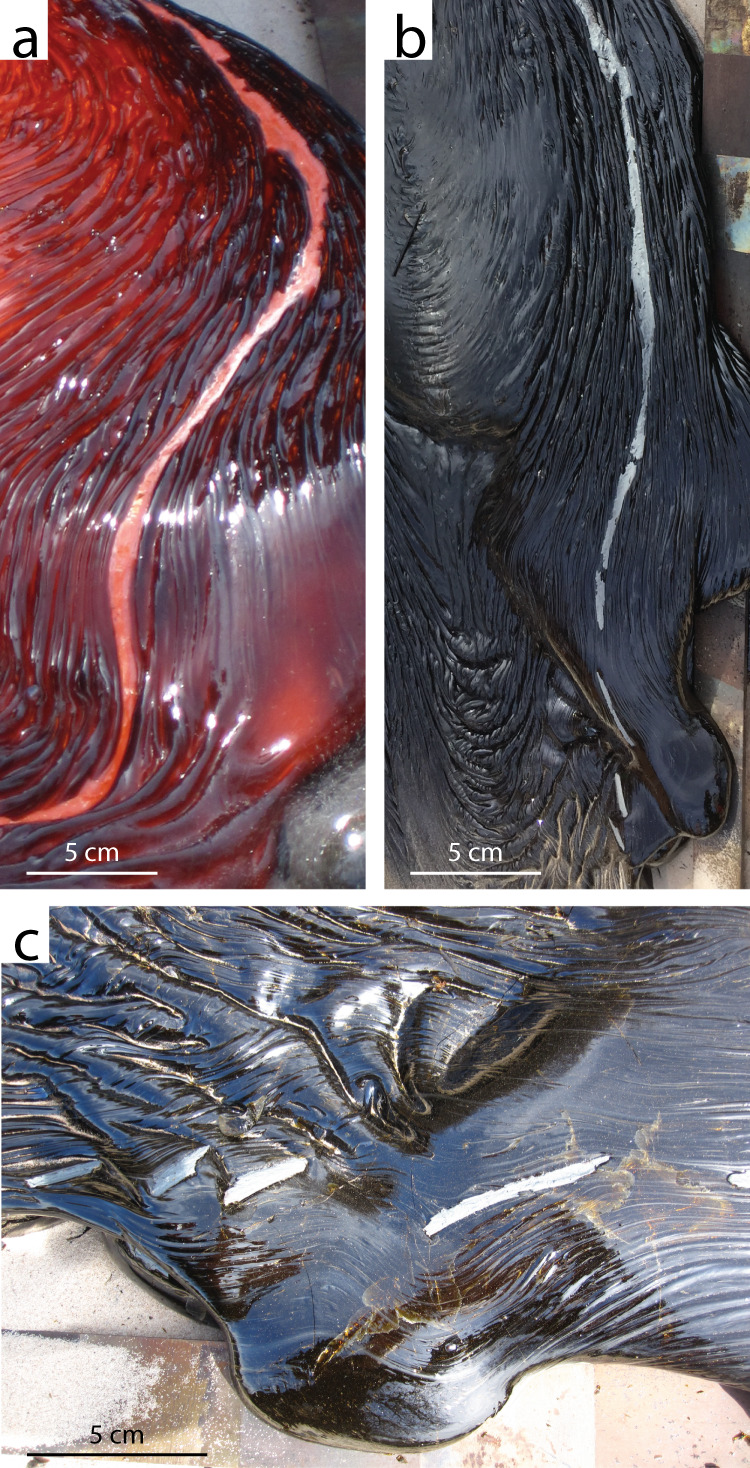
Surficial metallic flow morphology. **a** A small metallic flow (bright orange) on top of the silicate flow (dark orange), following the topography of the ropey silicate flow surface texture. **b** The aspect of the metallic flow (silver) in **a** after cooling. **c** Close up of **b**: note how the metallic ribbon is dismembered *en échelon* downstream.

**Fig. 5:**
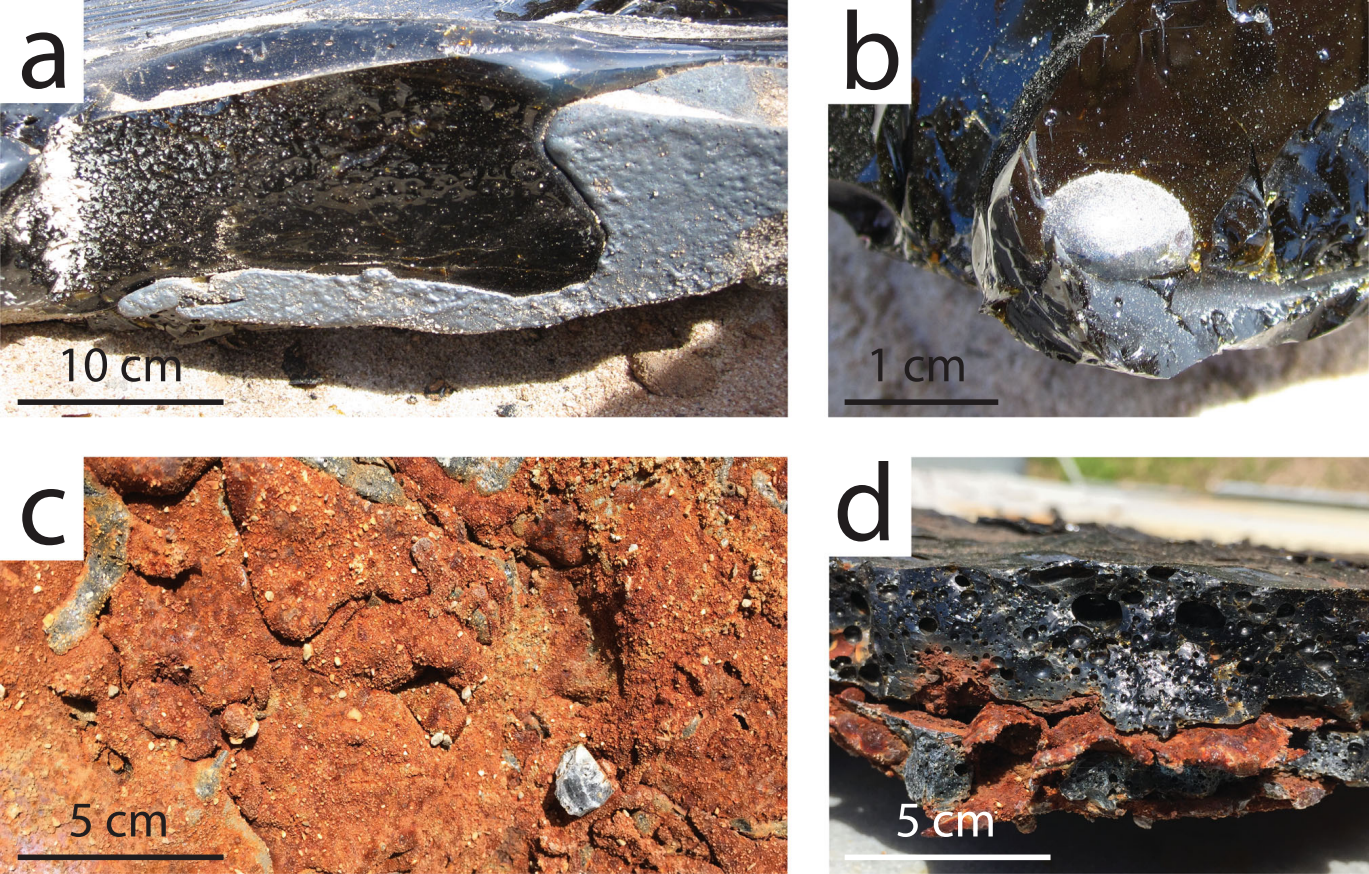
Flow contacts. **a** Sharp basal contact between the metallic (bottom, silver) and silicate (top, black) flow units. **b** Isolated metallic droplet within the silicate flow. **c** Base of the metallic flow (rusty color due to oxidation), showing rip-up clasts and erosional scours. **d** Metallic cross-cutting feature (rusty color due to oxidation) within the silicate flow.

The silicate flow body has a typical sheet morphology with a ropey surface (Fig. 2). The metallic flow front extends past the silicate flow front (Fig. 2). The first two violent breakouts of metal from the silicate flow front resulted in low-relief, highly braided, not fully cohesive metal channels (Fig. 3a) whose morphology evolved rapidly, whereas the latest, gentle breakout was an extrusion that produced a coherent, lumpy metal body (Fig. 3b). Volumetrically minor, dismembered metallic melt streams are found over the silicate flow, and follow its ropey texture (Fig. 4). Post-experimental flow dissection revealed a thin (about 1 cm), braided, mostly (but not totally) cohesive metallic flow body underlying the silicate flow. The contact between the silicate and metallic flows, preserved and sampled post- emplacement, is sharp (e.g., Fig. 5a). Part of the molten metal separated from the main metallic flow body, forming isolated small (cm-size) metallic droplets (Fig. 5b). There are clear signs of turbulent flow: rip-up clasts of sand substrate, basaltic and metallic fragments, and erosional topography (Fig. 5c). Locally, the metallic melt forms small cross-cutting features across the silicate flow (Fig. 5d): the metallic melt seeps through cracks in the developing upper silicate flow crust, and once it reaches the still molten silicate flow core it keeps flowing within it, remaining horizontally stratified above the basal silicate crust.

### Flow textures and compositions

Despite complex mingling, silicate and metallic flows remain physically segregated along sharp contacts (e.g., Fig. 5a). Starting material composition and post-experimental flow compositions (both silicate and metallic units) are presented in Table 1. Standard deviation is less than 1 wt% for each analyte. We typically observe a progressive enrichment in silica content and depletion in iron content with every successive silicate flow emplaced during a week-long experimental series. The final pour is the only one consisting of both a silicate and metallic flow. The starting material has a silica content of 54.13 wt% and an iron oxide content of 9.48 wt%. The last silicate flow has a silica content of 57.14 wt% and an iron oxide content of 6.49 wt%.

**Table 1:** Geochemical composition (wt%) of starting material and flow units.

	SiO_2_	TiO_2_	Al_2_O_3_	Na_2_O	MgO	K_2_O	CaO	MnO	FeO	NiO	P_2_O_5_	Cr_2_O_3_	Total
Starting material	54.13	1.89	15.78	2.80	6.36	0.86	6.37	0.19	9.48		0.15		100.00
Silicate flow	57.14	1.88	15.69	1.97	6.10	0.90	9.38	0.20	6.49	0.00	0.05	0.01	99.81
	**Si**								**Fe**	**Ni**	**P**	**Cr**	
Metal dendrites	0.35								96.45	0.81	0.75	0.02	98.37
Metal matrix	0.02								87.04	0.87	10.91	0.04	98.87

**Fig. 6:**
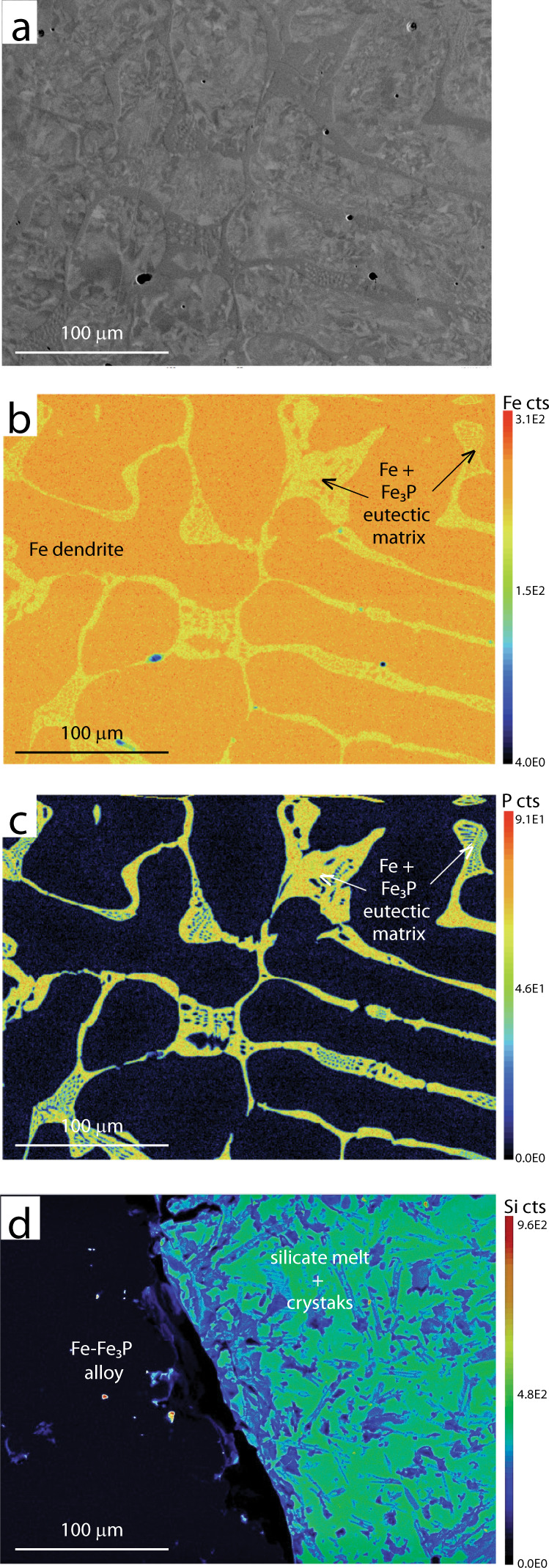
Metallic flow micro-textures. **a** BSE image of the metallic flow hypoeutectic alloy. **b** EDS iron map of **a**. **c** EDS phosphorus map of **a**; **d** EDS silicon map of the metallic (left)-silicate (right) flow contact.

The silicate flow is glassy (e.g., Fig 2a). However, in proximity of the metallic flow, it contains abundant acicular crystals (Fig. 6d).

The metallic flow (Fig. 6a) is composed of two phases: dendrites and a matrix. Dendrites are the volumetrically predominant phase, representing 78 vol% of the metal flow. The dendritic phase composition (Fig. 6b) is on average 96.45 wt% iron, with minor nickel, phosphorus, and silica, whereas the matrix composition (Fig. 6c) is on average 87.75 wt% iron and 10.13 wt% phosphorus, with minor nickel.

## Discussion

**Fig. 7:**
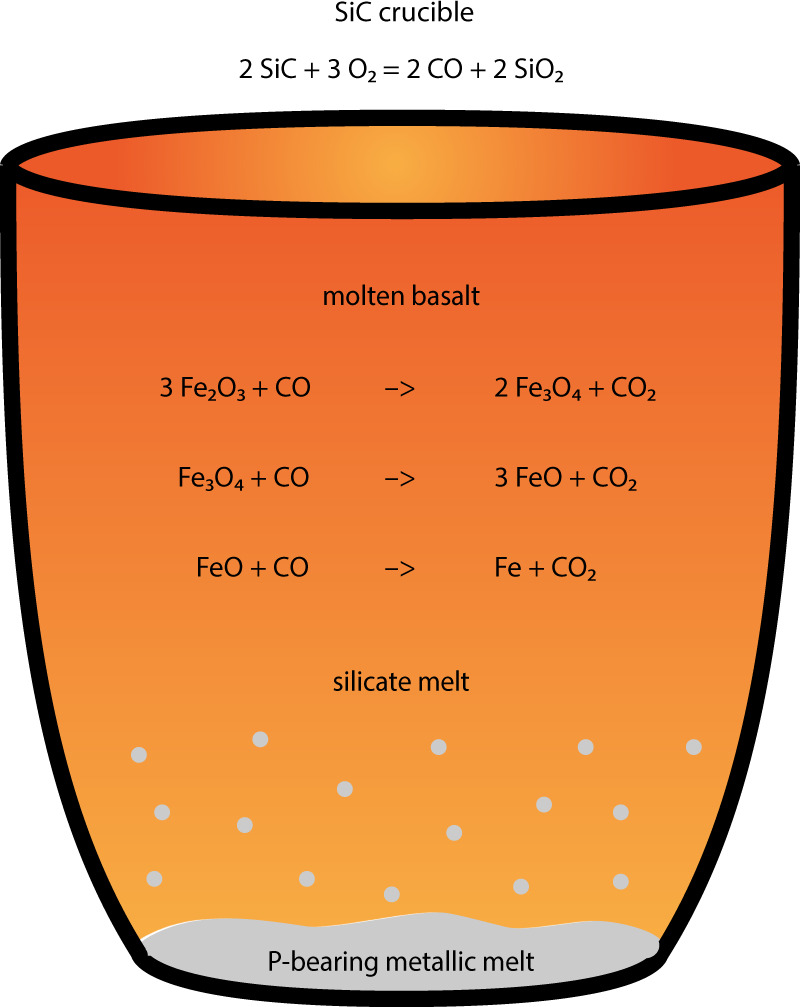
Metallic melt segregation. Schematic illustration of the spontaneous segregation of metallic melt from silicate melt in the experimental furnace. The silicon carbide crucible degrades into silica and carbon monoxide. The carbon monoxide reduces the basaltic melt. This causes part of the ferric and ferrous iron contained in the basaltic melt to reduce all the way to metallic iron and to precipitate and pool at the bottom of the crucible. Phosphorous, which becomes siderophile under reducing conditions, partially migrates into the metallic melt.

The experimentally observed metallic melt forms by segregation from the parent silicate melt (Fig. 7), due to the Si-Fe miscibility gap^16,17^. This process is oxygen fugacity-driven. The atmosphere within the experimental furnace is highly reducing. This is due to the thermal degradation of the silicon carbide crucible, following the reaction 2SiC + 3O_2_ → 2CO + 2SiO_2_, which onsets at temperatures as low as 450 °C, far below the furnace standard operating temperature (>1300 °C). The presence of abundant CO results in the reduction of part of the iron contained in the silicate melt to metallic iron at temperatures around 1250 °C. Being denser, the metallic iron melt precipitates and pools at the bottom of the crucible. This process is analogous to industrial iron smelting, with the difference that in industry CO is purposefully pumped into the source ore and limestone is added as a fluxing agent, resulting in the production of a slag. It is therefore clear that the iron that is observed flowing comes from the starting silicate material itself. In addition, some phosphorus is also extracted from the basalt. This is due to the fact that as redox conditions become more reducing, phosphorus becomes increasingly siderophile^18^.

At the Syracuse Lava Project facility, the crucible is only partially emptied for each standard experiment. Iron from many incremental starting material (basalt) additions to the mix can therefore accumulate, and metallic iron continues to be extracted from all material present in the furnace. This dense metal pools at the bottom of the crucible, remains segregated from the overlying, less dense basaltic melt, and is not emplaced during standard pours. When the crucible is emptied completely at the end of each experimental cycle, the extracted metal flows out of the crucible. The metallic and silicate flows mingle but do not mix during emplacement (e.g., Fig. 5a).

**Fig. 8:**
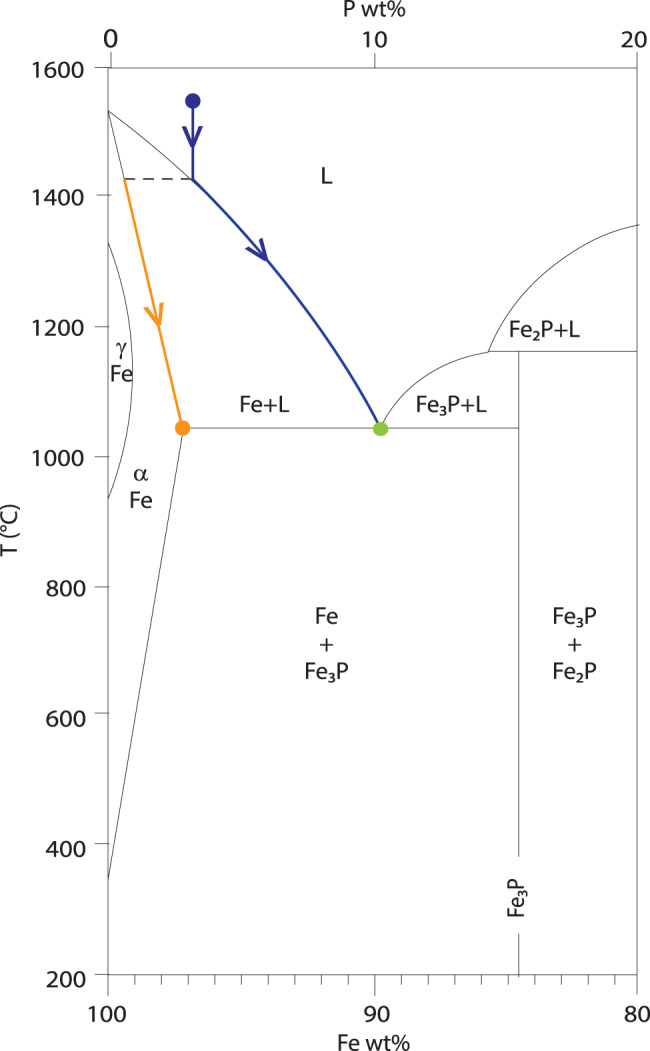
Metallic melt crystallization history. Fe–P binary phase diagram^40^ illustrating the temperature-composition evolution of the metallic melt: blue dot represents initial liquid composition; blue arrows represent liquid composition evolution; orange arrow represents crystallizing solid (dendrites) composition evolution; green dot represents eutectic matrix crystallization. Adapted by the permission from Springer Nature Customer Service Centre GmbH: Springer IRON-binary phase diagrams by Ortrud Kubaschewski von Goldbeck, ^©^Springer-Verlag Berlin Heidelberg (1982).

The crystallization history of the metallic flow is reconstructed from its measured composition and observed texture. We estimate the original metallic melt composition to be about 97 wt% iron and 3 wt% phosphorus based on the volumetric proportions and chemical compositions of the dendritic and matrix phases, and illustrate its idealized crystallization history in the Fe–P binary diagram (Fig. 8). As the metallic melt cools down, it reaches the liquidus and starts crystallizing ferrite (Fe) dendrites. The residual liquid becomes progressively enriched in iron phosphide (Fe_3_P), until it reaches the eutectic. At that point, the remaining liquid solidifies into the iron-iron phosphide eutectic solid at constant temperature (1048 °C). The final product is a Fe–P hypoeutectic alloy. Note that our metallic melt is not a pure Fe–P binary solution (Fig. 8), but rather contains Si, Ni, and Cr traces (Table 1). As a result, the matrix has a normalized iron content of 88.9 wt%, as opposed to the ideal eutectic iron content of 89.8 wt%. Likewise, the ferrite dendrites have a normalized iron content of 99.2 wt%, instead of 100 wt%. Remarkably similar textures and compositions have been produced experimentally by Török and Thiele^19^ while attempting to reproduce the medieval bog iron smelting process.

The intrinsic properties of the Fe–Fe_3_P alloy are well known^20^, but prior to this study, there were no available scientific data on the viscosity, dynamics, and morphologies of its surface flow. Industrial metal pours from steel mills are visually, qualitatively similar to our experimental metallic flows, and future analyses of their flow behavior might prove them to be useful analogs.

Initially, the silicate flow emplacement proceeds autonomously (Fig. 1a). Later, the metallic liquid joins the flow (Fig. 1b). The metallic liquid has a much higher density but much lower viscosity than the silicate melt (at the same T = 1150–1200 °C). The metallic flow density is 6950 kg/m^3^, about 2.8 times denser than the silicate flow. The silicate flow density is 2500 kg/m^3^, typical of basaltic materials. Because of this, most of the metallic material flows underneath the silicate flow resulting in a series of inflation bulges traveling at 0.18 m/s. Through Jeffrey’s equation^21^, the silicate lava viscosity is estimated to be around 153 Pa s; the metallic lava viscosity is estimated to be 1 Pa s, two orders of magnitude lower (Table 2). Very rapid, locally turbulent flow occurs in the metallic flow, resulting in physical mingling along the interface between the metallic and silicate flows (e.g., Fig. 5a, b). Finally, the metallic flow emplacement concludes independently of the silicate flow. The metallic flow rapidly outpaces the basaltic flow, reaching the flow front and propagating beyond it with distinct, largely turbulent dynamics (Fig. 5c).

**Table 2:** Flow parameters.

	Slope	Thickness	Density	Velocity	Viscosity	Surface tension
Silicate	12°	0.06 m	2500 kg/m^3^	0.04 m/s	153 Ps s	0.35 N/m
Metallic	12°	0.01 m	6980 kg/m^3^	0.41 m/s	1 Pa s	1.92 N/m

Viscosity is calculated through Jeffreys’ equation^21^; surface tension is based upon literature data: basalt at the liquidus from McBirney and Murase^37^; iron at the melting point from Kasama et al.^38^, and is to be taken as a maximum value, as alloys have a lower surface tension^39^.

Scaling experimental systems to natural ones is challenging, yet fundamental. The large-scale Syracuse experiments provide several advantages in this respect: (1) the use of natural materials eliminates the need of material properties scaling; (2) thermal scaling is also unnecessary, as experimental and natural temperatures match; (3) experimental flows are large enough to represent natural flow lobes in their own right prior to any scaling.

However, in order to establish the relevance and applicability of our experimental observations and results to full-size natural scenarios, we verify that experimental and natural flows are dynamically similar. Dynamic similarity ensures that the relative contribution of the various forces acting on the experimental and natural flows is the same, regardless of their absolute value. For lava flows, the relevant forces are inertia, viscosity, buoyancy, and surface tension. The balance among these forces is expressed through dimensionless groups, in particular the Reynolds number (Re), i.e., the ratio of inertial to viscous forces, and the Eötvös number (Eo), i.e., the ratio of buoyancy to surface tension forces. For Re above about 2000 the flow is turbulent, below it is laminar; for Eo above 40 surface tension is negligible^22^, below it matters. Dynamic similarity is verified by comparing the values of these dimensionless group, which should fall within the same field. Re values for our experiments are ~1 for the silicate flow (laminar flow regime) and ~5700 for the metallic flow (turbulent flow regime).

Natural flows on Earth will only differ in their characteristic length dimension (thickness for Eo and length for Re), and as much as a 100× or even 1000× size increase will not affect their Re regime. Eo values for our experiments are ~250 for the silicate flow (negligible surface tension effect, as also found by Griffiths^23^) and ~4 (non-negligible surface tension effect) to ~70 (negligible surface tension effect) for the metallic flow. Natural flows will only differ in their characteristic length dimension in this case as well. Let us consider a natural lava flow 100× larger (thicker, longer, and wider) than the experimental flow. A size increase will not affect the silicate flow, as surface tension will become more and more irrelevant. A size increase smaller than 4× will keep most of the metallic flow in the Eo regime where surface tension matters, whereas any further size increase will push the metallic flow into the Eo regime where surface tension has a negligible effect. The way in which surface tension affects the metallic flow in our experiment is by promoting a braided morphology and favoring droplet separation from its ~1 cm thin front (Figs. 1e, f and 3a). However, at a later stage of our experiment, we also observe coherent metallic flow lobes (Figs. 1g, h and 3b): being ~4–5 cm thick, they fall within the Eo regime that does not feel the effect of surface tension. In larger natural flows, we still expect to observe both regimes, with the high Eo regime dominating everywhere except for the very front of the flow.

As we expect ferrovolcanism to occur or have occurred on other planetary bodies as well, we extend our dimensional analyses to surface conditions on Mars and Psyche as well by considering their different gravitational acceleration, all other flow parameters remaining equal (flow size at 100× of the experimental flow). We find that both Re and Eo number regimes remain unchanged for all planetary bodies considered. We note however that gravitational acceleration, as well as surface temperature and atmospheric density, does affect other flow parameters in complex ways, which we do not account for here. We further note that oxidation conditions vary across the solar system, with Mars and Psyche having more reducing surface conditions than Earth. Reduced melts are less viscous than oxidized ones^24^, thus we expect flows to be more turbulent on Mars and Psyche than on Earth. However, the difference is not thought to be sufficient to cause a change in flow regime.

Further details on dimensional analyses can be found in Supplementary Material 1.

Our findings have important applications in planetary research, as potentially ferrovolcanic worlds are discovered and explored.

Owing to its composition, 16 Psyche is a prime candidate to have hosted type I ferrovolcanism. Our experiments provide a starting point to interpret its surface morphology once it becomes known. In fact, in late experimental phases (Fig. 1d–h), the emplacement of the metallic melt proceeded largely independently from that of the silicate melt: the frontal part of the flow was entirely metallic, and thus provided insight relevant to type I ferrovolcanism. For example, our results strongly suggest that ferrovolcanic topography will likely be characterized by minimal topographic relief. Due to its ultra-low viscosity, we expect that ferrovolcanic magma will at most build extremely low-aspect ratio volcanic landforms, by successive accumulation of long, thin, sheet flows. This agrees with the hypothesis of a lack of proper ferrovolcanic edifices on Psyche, brought forward by Johnson et al.^5^ based on the low viscosity and low-gas content expected in ferrovolcanic magma.

Mars could have hosted type IIb ferrovolcanism instead. Like Earth, Mars is a rocky planet, and therefore silicate volcanism is expected, and its associated landforms have indeed been observed. However, in addition to silicate volcanism, ferrovolcanism may have occurred on Mars, perhaps more commonly than on Earth due to crustal composition differences between the two planets. Notably, the martian crust is significantly more iron-rich than the terrestrial crust, as well as marginally more phosphorus-rich^25^, with both of these characteristics favoring Fe–Si liquid immiscibility^12^. It is therefore possible that type IIb ferrovolcanic flows may have been routinely emplaced on Mars while the planet was still volcanically active. We expect that Martian type IIb ferrovolcanic flows would look similar to the mixed silicate-metallic experimental flow that we produced in this study. Specific geomorphological features that would confirm the ferrovolcanic genesis of some Martian lava flows include inflated silicate flows with thinner, lumpy, and braided metallic tendrils emerging from the flow front and, occasionally, sides. It is also possible that the braided channel network underlying the silicate flow formed by the metallic melt would be partially or entirely exposed as a result of post-emplacement differential erosional processes.

Our experiments further suggest that the very different morphologies of metallic and silicate flows (resulting from their contrasting flow dynamics) may be used to differentiate between these different flow types. A higher resolution than those currently available through orbiters’ remote sensors might be necessary to operate this distinction; however, imagery acquired by landers could certainly be used to that end.

On Earth, Kiruna-type iron deposits^26^ are a strategically important resource whose genesis is still debated. Keller et al. (2019)^12^ proposed that the magnetite-apatite deposits of El Laco volcano (Chile), may be explained by the generation and extrusion of Fe-rich magmatic liquid. These types of deposits may be small-scale examples of mixed mode, silicate-metallic volcanism (ferrovolcanism type IIb) that could occur on other planetary bodies. This study provides the basis to investigate the emplacement dynamics of surface flows of such melts.

Finally, beyond their direct relevance to ferrovolcanic flows, the experimental flows described in this study may shed light on the emplacement of mixed rheology flows in general. Although most volcanic eruptions will not encompass such contrasting materials as iron and basalt, these can be taken as endmember scenarios which can nonetheless inform our understanding of the emplacement dynamics of immiscible, mingled magmas with different physical properties, such as the classic bimodal basalt-rhyolite terranes of Earth^27^ and the basalt-sulfur flows of the Jovian moon Io^28^.

The coeval emplacement of experimental immiscible silicate and metallic flows of contrasting densities and viscosities was observed and analyzed for the first time here, in an experimental setting. The distinct physical properties and flow dynamics of basaltic and iron-rich metallic flows have implications for the understanding of ferrovolcanism on planetary bodies of the solar system and mixed mode coeveal flows on Earth.

The main findings of our study can be summarized as follows:

1. Silicate and metallic flows emplaced coevally interact dynamically, as evidenced by meso-scale mingling textures.
2. Coeval silicate and metallic flows both retain their own distinctive overall morphological features: silicate flows form coherent, ropey sheets, whereas metallic flows display two different morphologies: (1) lumpy and cohesive and (2) braided and not fully cohesive.
3. Metallic flows are highly turbulent; therefore, their emplacement dynamics cannot be simply inferred by analogy with laminar silicate flow dynamics, and neither can their final emplacement morphology.

## Methods

### Experimental setup

The Syracuse University Lava Project represents the only large-scale experimental facility of its kind in the world. It has the capability of producing volume-limited lava flows of comparable size to individual natural pāhoehoe flow lobes (1-2 m) under controlled conditions. The gas-fired Gasmac tilting furnace was originally designed for bronze foundry work, and was reconditioned to work with basalt. The furnace assembly has been configured to allow both temperature (900–1250 °C) and pouring rate (100–700 cm^3^/s) to be controlled. The silicon carbide crucible can hold up to 450 kg (or 0.18 m^3^) of basaltic melt at one time^29^. Temperature, effusion rate, substrate material, and slope can be varied according to experimental necessities. The setup includes both longitudinal and vertical scale bars within the viewing area to allow quantitative data extraction from acquired videos. So far, the Syracuse Lava Project has enabled a wide range of experimental studies of flow dynamics^30^, flow rheology^31^, flow morphology^32,33^, lava–ice interactions^34^, and barrier efficiency^35^.

For the study presented in this paper, the molten rock was held at the target temperature for several hours to ensure a volatile-free melt. Lava was then poured through a metal chute and onto a slope covered in quartz sand. The flow was observed and recorded using vertical and oblique high-definition video cameras. Particle image velocimetry on the experimental footage was performed on the experimental footage through the Kinovea software, as detailed in Farrell et al.^32^, to determine flow velocity. Flow core temperature was measured with a K-type thermocouple inserted in the active flow core. After the flow cooled down, portions of the flow were sectioned, and meso- and micro-structures were investigated.

### Starting material

The starting material used for these experiments is 1.1 Ga, high-Fe tholeiitic basalt (Table 1) from the Chengwatana Formation of the Mid-Continent Rift^36^, crushed into cm-size pieces. This material has been used in numerous previous experimental flows and is well characterized in terms of composition and flow parameters^30,32^. Both the silicate and metallic flows result from this material, as detailed in the “Discussion” section, and the metallic flow is studied for the first time.

### Textural and chemical analyses

In order to examine the texture of both the silicate and metallic flow units, as well as their contact, the flow was sectioned and examined at the hand sample, mesoscopic scale (cm-dm). In addition, thin sections were made perpendicular to the contact at a frontal breakout location in order to study microscopic scale (μm-mm) features. Backscattered electron images and elemental maps of representative portions of each thin section were acquired at 50x magnification. Elemental maps and analysis of experimental products were used to determine the composition of the silicate and metallic melts. All analyses and imaging were conducted at the Syracuse University Electron Microscope Laboratory on a Cameca SXFive Electron Microprobe. Elemental maps were further processed with the free software JMicroVision to establish phase proportions.

## Supplementary information

Supplementary Information

Description of Additional Supplementary Files

Supplementary Movie 1
