# Supplementary material for: Imagining and constraining ferrovolcanic eruptions and landscapes through large-scale experiments
Source: Nat Commun. 2021 Mar 17;12:1711. doi: 10.1038/s41467-021-21582-w (PMC7969621; doi:10.1038/s41467-021-21582-w)

		viscosity Pa s	surface tension N/m	density kg/m3	velocity m/s	thickness m	length m	g	Eo	Re
Experiment	***silicate***	*153*	*0.35*	*2500*	*0.04*	*0.06*	*1*	*9.81*	*252*	*1*
**Earth**	**silicate**	153	0.35	2500	0.04	6	100	9.81	2522571	131
**Mars**	**silicate**	153	0.35	2500	0.04	6	100	3.71	954000	131
**Psyche**	**silicate**	153	0.35	2500	0.04	6	100	0.06	15429	131
Experiment	***metallic***	*1*	*1.92*	*6980*	*0.41*	*0.01*	*1*	*9.81*	*4*	*5724*
Experiment	***metallic***	*1*	*1.92*	*6980*	*0.41*	*0.045*	*1*	*9.81*	*72*	*5724*
**Earth**	**metallic**	1	1.92	6980	0.41	1	100	9.81	35663	572360
**Mars**	**metallic**	1	1.92	6980	0.41	1	100	3.71	13487	572360
**Psyche**	**metallic**	1	1.92	6980	0.41	1	100	0.06	218	572360

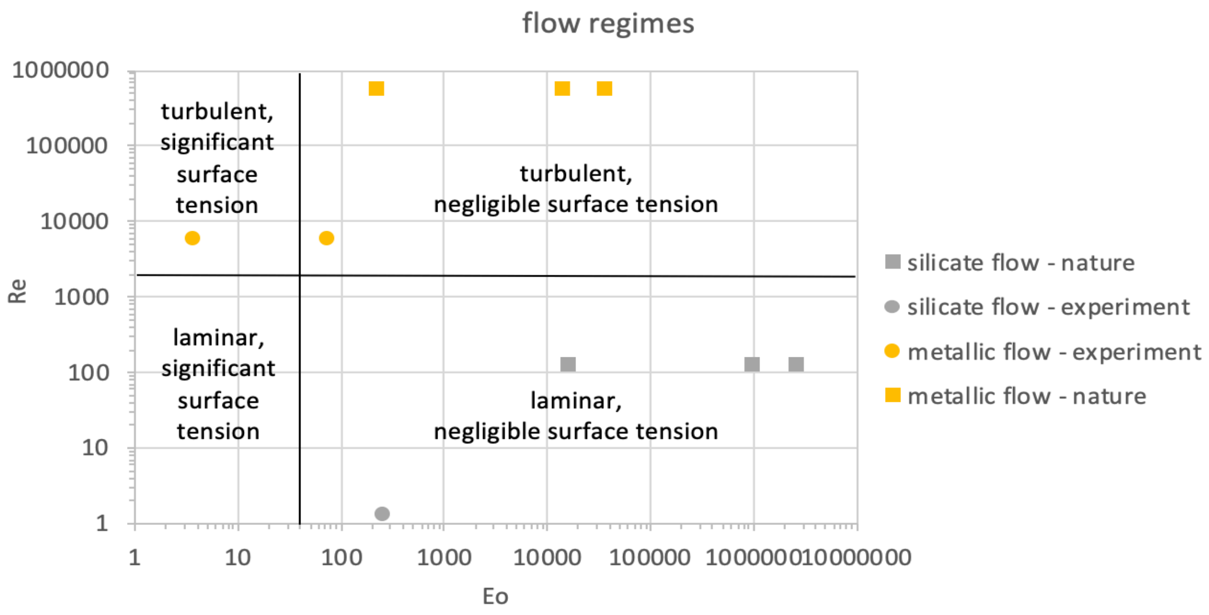

Supplement: Supplementary file 1 — Supplementary Information [file 41467_2021_21582_MOESM1_ESM.pdf]
