# Supplementary material for: Imagining and constraining ferrovolcanic eruptions and landscapes through large-scale experiments
Source: Nat Commun. 2021 Mar 17;12:1711. doi: 10.1038/s41467-021-21582-w (PMC7969621; doi:10.1038/s41467-021-21582-w)
Supplement: Supplementary file 2 — Description of Additional Supplementary Files [file 41467_2021_21582_MOESM2_ESM.pdf]

Description of Additional Supplementary Files

File Name: Supplementary Movie 1
Description: Experimental ferrovolcanic flow emplacement (flow 170410).
